# Genetic-parameter estimation of milk yield in White Maritza sheep breed using different test day models

**DOI:** 10.5194/aab-66-253-2023

**Published:** 2023-09-15

**Authors:** Petya Zhelyazkova, Doytcho Dimov, Sreten Andonov

**Affiliations:** 1 Department of Animal Sciences, Agricultural University, Plovdiv, Bulgaria; 2 Department of Animal Breeding and Genetics, Swedish University of Agricultural Sciences, Uppsala, Sweden; 3 Institute of Animal Biotechnology, Faculty of Agricultural Sciences, Saints Cyril and Methodius University, Skopje, North Macedonia

## Abstract

The aims of this study were to estimate the genetic parameters of the test day milk yield (TDMY) of the White Maritza sheep breed population and to choose the most appropriate linear models for genetic-parameter estimation of test day milk yield. The White Maritza sheep breed is a multipurpose native sheep breed in Bulgaria. Test day milk yield data were collected from 1992 to 2015 (24 years). Milk yield recordings were made in 18 flocks according to the AC method (official milk recording by ICAR regulations). The database includes 8768 test day milk yield records belonging to 987 ewes. The pedigree file includes 1937 animals. Nine test day models (TDMs) were formulated and tested for the estimation of the genetic parameters of milk yield. The first three models were repeatability models (REP models), the second three were random regression models (RRMs), and the last three models were also random regression models with an added Ali and Schaeffer regression to describe the lactation curve using first-, second- and third-order polynomials. The average TDMY was 764.47 mL. There were no significant differences in the values of heritability ( $h^{2}$ ) calculated by the three REP models: REP1 0.355 ± 0.060, REP2 0.344 ± 0.047 and REP3 0.347 ± 0.060. The same applied to the repeatability coefficients, which, for the three REP models, were 0.384 ± 0.065, 0.376 ± 0.051 and 0.378 ± 0.065, respectively. Based on REP model 1, three models with random regression RRM1, RRM2 and RRM3 were constructed, which is associated with the use of first-, second- and third-order polynomials (for the random effects of both the animal and the permanent environment). The trajectories of $h^{2}$ calculated by the three RRMs were not similar and demonstrated some differences, both at the beginning and in the middle of the milking period. The RRM with third-order polynomials demonstrated more genetic diversity until the 165th day of lactation, but Akaike information criterion (AIC), Bayesian information criterion (BIC) and log-likelihood (LogL) estimates were higher. The regression models with first- and second-degree polynomials were insufficient to reveal genetic diversity to a higher degree than REP model 1. The trend in the trajectories of $h^{2}$ calculated by the three random regression models with Ali and Schaeffer regression models (ASRMs) was similar to that of random regression models without the Ali and Schaeffer regression incorporated. Although the noted advantages of the random regression models revealed, to a greater extent, the genetic diversity of test day milk yield, AIC, BIC and LogL estimates indicated that repeatability models achieved a better balance between complexity and fitness and a smaller prediction error compared to random regression models.

## 1 Introduction

The high demand for sheep milk as a raw material for the production of different dairy products over a long-term period will form a favorable market environment for dairy and dual-purpose sheep breeds in the countries of the European Union (EU). There is growing interest in the breeding of native or improved sheep breeds with good milk production in countries such as Slovenia, Slovakia, the Czech Republic, Croatia and Spain. For the genetic improvement of milk yield in many native sheep breeds in the EU, a number of authors developed, tested and implemented various test day models (TDMs) to estimate genetic parameters in sheep (Serrano et al., 2001; Oravcová et al., 2005; Oravcova, 2007; Gutierrez et al., 2007; Oravcova and Peskovicova, 2014; Komprej et al., 2013; Špehar et al., 2020).

The milk yield of sheep is an important trait for selection in the breeding programs of prospective native Bulgarian sheep breeds. The production systems in which the White Maritza sheep breed and other sheep breeds in Bulgaria which are suitable for milk production are kept have two distinctive features. The first distinguishing feature is that, in all native breeds in the early phase of lactation, lambs suckle from their mothers – that is, there is a suckling period, where the duration for White Maritza sheep is 67.5 d (Dimov, 2011), for the synthetic population of dairy sheep it is 63.5 d (Zhelyazkova et al., 2014), and for Patch-faced Maritza sheep it is 62.5 d (Zhelyazkova and Dimov, 2022). The second distinguishing feature is that, during the second half of pregnancy and the first months of lactation, the sheep are reared indoors, and during most of the milking period, they graze. Therefore, when estimating the genetic parameters and breeding values in sheep in relation to milk yield, this specificity of production systems must be taken into account in the models used.

The best linear unbiased prediction (BLUP) procedure and related animal models have become the standard for the genetic evaluation of the traits associated with milk productivity in sheep (Barillet et al., 1992b, 2001; Legarra and Ugarte, 2004; Jimenez and Jurado, 2006, etc.). Until 2001, the estimation of genetic parameters for milk yield in sheep was the basis of the lactation model (El-Saied et al., 1998a, b; Portolano et al., 2001). On the basis of test day measurements, milk yield in standard or whole lactation was calculated and used further in genetic analyses (Kovac et al., 2001). Komprej et al. (2009) noted that lactation models for estimating genetic parameters in sheep have some deficiencies. Breeding values are predicted on the basis of only one record (milk yield after weaning of the lambs to the end of lactation) per animal; therefore, the individual test day records were not adjusted for specific environmental effects, and genetic evaluation could be performed only when lactation was completed. For these reasons, the lactation model was replaced by the test day model (TDM). In cattle, genetic evaluations based on test day yields offer many advantages over those based on 305 d lactations, including better modeling of the factors affecting the yields; the fact that there is no need to extend records; and, possibly, greater accuracy of evaluations (Ptak and Schaeffer, 1993). This approach was later accepted in dairy sheep breeding.

Initially, test day models (TDMs) were developed and used as repeatability models in which dairy records of the test day were treated as repeated measurements of the same trait (Barillet and Boichard, 1994; El-Saied et al., 1998; Serrano et al., 2001; Oravcová et al., 2005; Komprej et al., 2009). However, the use of repeatability test day models is associated with some assumptions that variations and genetic correlations of the unity among yields at different stages of lactation are constant and that all animals have a standard lactation curve – that is, differences in lactation persistence between animals are ignored (Ptak and Schaeffer, 1993; Komprej et al., 2013). Soon after the introduction of the repeatability model, the random regression models were introduced; in the last 2 decades, these became a model of choice for genetic-parameter estimation in animal breeding (Komprej et al., 2013).

Random regression models can typically be used when a trait is expressed repeatedly, e.g., over time or in different environments. In that case, the effect changes gradually along a trajectory of time or of some other continuous variable (Van der Werf, 2003). In the random regression model, the individual measurements of TDMYs are considered to be different traits. In addition, the random regression model makes it possible to take into account the different shape of the lactation curve in different individuals by including random regression coefficients for each animal (Jamrozik and Schaeffer, 1997). Comparing regression models with repeatability models, Andonov et al. (2013) found out that the genetic merit in the Norwegian goat population could be predicted most precisely with random regression models.

In Bulgaria, TDMs still have limited application for the assessment of the effects of genetic parameters and the environmental on the milk yields of sheep of different breeds and populations. A repeatability model was used by Krastanov et al. (2018) to analyze genetic variance (additive, dominant and epistatic) in an experiment in a flock of Bulgarian dairy synthetic population (BDSP) sheep. The same authors used the same database to assess the genealogical lines of the flock of the Agricultural Institute in Shumen on the basis of TDMYs, treating the individual measurements as separate traits, i.e., using a random regression model (Stancheva et al., 2017). So far, at the population level in Bulgaria, there are no applications of TDMs for the assessment of the genetic parameters and breeding values in sheep and goat breeds. Although the pedigree book and the milk recording of the White Maritza sheep breed were started in 1991, no genetic analysis of the milk yield has been performed so far.

The White Maritza sheep breed is a multipurpose lowlands-native sheep breed in Bulgaria, and in recent years, the breed has spread to some semi-mountainous regions. Good milk yield and prolificacy (Dimov, 1998), good growth rates of weaned lambs (Stoichev et al., 2015) and also a high live weight of mature sheep (Dimov, 2011) make this native sheep breed competitive among other native breeds in the country. The White Maritza sheep breed has good potential for milk yield; this is an important trait and should be used in a new breeding program, which should include modern genetic estimations. Given the small population size of the White Maritza sheep breed in milk-recording procedures over the years, the selection for milk productivity has been based on the trait of milk yield for the milking period following comparisons and evaluations. This selection mainly follows two pathways: mother–son or mother–daughter. This is a simple method of selection, and it is no longer sufficient because environmental effects are not taken into account.

The aims of this study were to estimate the genetic parameters of the test day milk yield (TDMY) of the White Maritza sheep breed population and to choose the most appropriate linear models according to the structure of the test day milk yield database.

## 2 Materials and methods

The data used for this analysis were provided by the breeding association of native Maritza sheep breeds (White Maritza and Patch-faced Maritza). For the purposes of this study, a test day milk yield database was structured. Data for test day milk yields were obtained from 1992 to 2015 (24 years). Milk yield recordings were made in 18 flocks. The mating system in all flocks was natural mating, and there was not artificial insemination during any of the years. Some limitations in the database were identified before the data were processed. The database included ewes with test day milk yields from 100 to 4000 mL, with suckling periods of 30 to 150 d. Each sheep in the database was required to have at least 3 test days. The number of lambs born was defined as a fixed effect with two levels. Due to the relatively small number of cases of ewes with triplets, these were included in the group of two-lamb sheep.

In order to take into account the effect of the long lambing campaign over the years, the lambing season was divided into three levels. The first level included ewes that were lambed in August, September, October and November. The second level included ewes that were lambed in December, January and February. The third level included ewes that were lambed in March, April, May and June. Thus, the seasonal effect was formulated, and this was combined with the effect of the year; the year–season effect was thus obtained in the models described below.

The continuity of the milk-recording procedure in the flocks for the studied period 1992–2015 differed by years. The number of flocks in which milk recordings lasted more than 7 years was four. Milk recordings in some flocks were usually maintained as a regular practice for 2 to 5 years. When constructing the linear models in this study, this specificity of the TDMY database was highlighted, where the milk recordings in the flocks were of different durations over the years. The comparatively long period (24 years) in which the data from the milk recordings were accumulated in a database and the short period of the continuity of breeding activity by the individual farmers (4.99 years) (Zhelyazkova et al., 2018) made it necessary to concatenate the flock–year–test-day factor.

The database included 8768 dairy records for the test day milk yield of 987 ewes bred in 18 flocks from 1992 to 2015. The data in Table 1 show that 6.32 % of the test day milk records belong to ewes that were lambed as yearling ewes. The highest proportion of dairy records on the test day belongs to ewes that are 2 and 3 years old (22.51 % and 21.61 %); 7.55 % of the dairy records in the database belong to ewes that are 7 to 10 years old. This fact, which is specific to the White Maritza sheep database, requires that one take into account the age at lambing when estimating the breeding values of ewes.

**Table 1:** Distribution of lactation records in classes according to age of lambing (rounded to whole years).

Age at lambing	Lactation	Share of age
(years)	records ( n )	groups (%)
1	128	6.32
2	456	22.51
3	438	21.62
4	381	18.81
5	284	14.02
6	186	9.18
7–10	153	7.55
Total	2026	100

Ewes of different ages are bred in all flocks of the White Maritza sheep breed, and there are no flocks that are formed by age.

Milk recordings were performed according to the AC method by ICAR regulations (Barillet et al., 1992a, and subsequent editions of the ICAR rules); the measurement was applied at one of the two or three daily milkings. The rule for the coverage of the maximum part of the milking period, regardless of its duration, was complied during the test day recordings over the years. As noted above, in the majority of the flocks, there is a long lambing campaign. After completing the milk recording in the milking period in the flocks of White Maritza sheep, it was found that different ewes have a different number of test days per lactation, ranging from 3 to 7 d. The pedigree file includes all animals with test day milk yield data and their ancestors, traced back to the generations to which their ancestors are known. All known relationships among ewes were included in the animal model. The pedigree information was analyzed by using the PEDIG program (Boichard, 2002). The database of the pedigree file contained 1937 ewes (Table 2). Only 1261 ewes had known two parents (dam and sire) for a total of 65.10 %. Sheep with a known sire but an unknown mother accounted for 119 animals, and sheep with a known mother but an unknown sire accounted for 26 animals. A total of 531 (27.41 %) sheep had two unknown parents (base animals).

The pedigree file contained 169 sires and 932 mothers. The pedigrees of all ewes with TDMY records constituted a total of 3038 animals that were identified as parents, grandparents, etc.

**Table 2:** Structure of pedigree file.

Pedigree structure	n	Percentage
		(%)
Pedigrees	1937	
Animals with known sire and dam	1261	65.10
Animals with known sire and unknown dam	119	6.15
Animals with known dam and unknown sire	26	1.34
Animals with unknown sire and dam	531	27.41
Sires	169	
Dams	932	
All animal in pedigree	3038	

The analysis of pedigree quality in White Maritza sheep breed sheep indicated that the average number of known generations was 2 for females and 2.14 for males; the average number of known generations for females and males was 2.05. Comparatively lowly known generations in terms of pedigree for female and male animals were the result of the different continuities of the breeding activity of sheep breeders participating in the milk-recording procedure and the filling-in of the pedigree book. The average number of traced ancestors was 15.62 for the female animals and 14.83 for the males; the average number of ancestors for the female and male animals in the database was 15.32 (Table 3).

**Table 3:** Pedigree quality in the White Maritza sheep breed database.

	Average for	Average for
	generation	ancestors
Female animals	2.00	15.62
Male animals	2.14	14.85
Average	2.05	15.32

Prior to the inclusion of the environmental factors affecting TDMY, their significance was tested using the general linear modeling (GLM) procedure of SPSS 19.0 for Windows (IBM Corp., 2010). Nine test day models (TDMs) were formulated and tested for the estimation of the genetic parameters of milk yield and for the breeding-value estimations of breeding animals of the White Maritza sheep breed. The first three models were repeatability models (REP models), the second three were random regression models (RRMs), and the last three models were also random regression models but with the addition of the Ali and Schaeffer linear regression to describe lactation curves (ASR; Ali and Schaeffer, 1987). The three tested models are described below as follows.

### 2.1 Repeatability models (REP models)

#### 2.1.1 Model 1

1 $$\begin{array}{rl} y_{ijklmn} & ={\mathrm{YS}}_{i}+\mathrm{DIM}3_{j}+{\mathrm{PAR}}_{k}+{\mathrm{LS}}_{l}+b_{1}(\mathrm{age}{)}^{2}\\ & +b_{2}(\mathrm{sp}{)}^{2}+{\mathrm{fytd}}_{m}+a_{n}+{\mathrm{pe}}_{n}+e_{ijklmn} \end{array}$$

In the above equation, $y_{ijklmn}$ is a vector of observations in terms of the TDMY for ewes n within the year–season of lambing class i , the stage of lactation j and the parity k with litter size l ; $b_{1}$ and $b_{2}$ are quadratic regressions of the age (age) and suckling period (sp) of ewes. Also represented is the flock–year–test-day factor m (fytd ${}_{m}$ ). YS ${}_{i}$ is the fixed effect of the year–season of lambing, with 67 classes; DIM3 ${}_{j}$ (representing days in milk) is the fixed effect of the stage of lactation, defined in 3 d intervals starting from day 30; PAR ${}_{k}$ is the fixed parity effect, accounting for seven classes; LS ${}_{l}$ is the fixed effect of litter size with two classes; $b_{1}(\mathrm{age}{)}^{2}$ is the fixed quadratic regression for age at lambing; $b_{2}(\mathrm{sp}{)}^{2}$ is the duration of the suckling period; fytd ${}_{m}$ is the random effect of flock–year–test-day m ; $a_{n}$ and ${\mathrm{pe}}_{n}$ are the random effects of the permanent environment of the animal and residual, respectively.

#### 2.1.2 Model 2

2 $$\begin{array}{rl} y_{iklmn} & ={\mathrm{YS}}_{i}+{\mathrm{PAR}}_{k}+{\mathrm{LS}}_{l}+b_{1}(\mathrm{age}{)}^{2}+b_{2}(\mathrm{sp}{)}^{2}\\ & +b_{3}(\mathrm{DIM})+{\mathrm{fytd}}_{m}+a_{n}+{\mathrm{pe}}_{n}+e_{iklmn} \end{array}$$

Model 2 is another specification capable of accounting for the lactation curve effect by replacing DIM3 in model 1 with the linear regression of DIM nested within parity. All other effects were kept the same.

#### 2.1.3 Model 3

3 $$\begin{array}{rl} y_{iklmn} & ={\mathrm{YS}}_{i}+{\mathrm{PAR}}_{k}+{\mathrm{LS}}_{l}+b_{1}(\mathrm{age}{)}^{2}+b_{2}(\mathrm{sp}{)}^{2}\\ & +b_{3}\left(\mathrm{DIM}/314\right)+b_{4}{\left(\mathrm{DIM}/314\right)}^{2}\\ & +b_{5}\mathrm{ln}\left(314/\mathrm{DIM}\right)+b_{6}{\left[\mathrm{ln}\left(314/\mathrm{DIM}\right)\right]}^{2}\\ & +{\mathrm{fytd}}_{m}+a_{n}+{\mathrm{pe}}_{n}+e_{iklmn} \end{array}$$

In the above equation, additional notation relative to that described for model 1 is $b_{3}$ , $b_{4}$ , $b_{5}$ and $b_{6}$ , which are fixed ASR coefficients across YS classes, and DIM, which is the day of lactation (33 to 314). The purpose of model 3 is to provide another explanation of the lactation curve derived by the Ali and Schaeffer fixed regression through the lambing sequence and to offer a replacement of the linear regression for the DIM indicated in model 2.

### 2.2 Random regression models (RRMs)

The REP model (model 1) was extended by adding Legendre polynomials of the first, second or third order for the random effects of both the animal and the permanent environment; this resulted in models denoted as RRM1, RRM2 and RRM3. The RRM models can be described as follows:

4 $$\begin{array}{rl} y_{iklmn} & ={\mathrm{YS}}_{i}+\mathrm{DIM}3_{j}+{\mathrm{PAR}}_{k}+{\mathrm{LS}}_{l}+b_{1}(\mathrm{age}{)}^{2}\\ & +b_{2}(\mathrm{sp}{)}^{2}+{\mathrm{fytd}}_{m}+\sum_{o=0}^{3}a_{on}Z_{on}\\ & +\sum_{o=0}^{3}{\mathrm{pe}}_{on}Z_{on}+e_{iklmn}, \end{array}$$

where an additional feature of this model is $Z_{on}$ , the polynomial o for DIM n , where o=0 , 1, 2, 3, shown in the same order for the effects of both the animal and the permanent environment; $a_{on}$ is the random regression coefficient of $Z_{on}$ for the genetic effect of the animal; pe ${}_{on}$ is a random regression coefficient of $Z_{on}$ for the permanent environmental effect, and $e_{ijklmn}$ is the residual effect.

At DIM, n additive genetic variance ( $\sigma_{a_{o}}^{2})$ , permanent environment variance ( $\sigma_{{\mathrm{pe}}_{o}}^{2}$ ) and heritability ( $h_{o}^{2}$ ) were calculated as follows:

5σao2=zo′Gzo,6σpeo2=zo′Pzo,

and

7 $$h_{o}^{2}=\frac{\sigma_{a_{o}}^{2}}{\sigma_{a_{o}}^{2}+\sigma_{{\mathrm{pe}}_{o}}^{2}+\sigma_{e}^{2}},$$

where $z_{o}$ is vector of polynomials in the model for DIM n , G is the (co)variance matrix for animal RR coefficients, P is the (co)variance matrix for permanent environment RR coefficients, and $\sigma_{e}^{2}$ is the residual variance. Note that model 1 is equivalent to the random regression model of order o=0 .

We now refer to Ali and Schaeffer regression models (ASR models).

8 $$\begin{array}{rl} y_{iklmn} & ={\mathrm{YS}}_{i}+{\mathrm{PAR}}_{k}+{\mathrm{LS}}_{l}+b_{1}(\mathrm{age}{)}^{2}+b_{2}(\mathrm{sp}{)}^{2}\\ & +b_{3}\left(\mathrm{DIM}/314\right)+b_{4}{\left(\mathrm{DIM}/314\right)}^{2}\\ & +b_{5}\mathrm{ln}\left(314/\mathrm{DIM}\right)+b_{6}{\left[\mathrm{ln}\left(314/\mathrm{DIM}\right)\right]}^{2}\\ & +{\mathrm{fytd}}_{m}+\sum_{o=0}^{3}a_{on}Z_{on}\\ & +\sum_{o=0}^{3}{\mathrm{pe}}_{on}Z_{on}+e_{iklmn} \end{array}$$

The REP model 3 (REP 3) is deployed by adding a linear regression to describe the lactation curve using first-, second- and third-order polynomials, for both permanent animal effects and permanent environmental effects. As a result, the models are described as ASRM 1, ASRM 2 and ASRM 3.

In all models, homogeneous residual variances were assumed similarly to in Andonov et al. (2007). The random animal genetic effects were assumed to have (co)variance structures proportional to the additive relationship matrix, whereas the repeated animal effects were unstructured. Estimates of variance components were performed on the basis of a single univariate REP model using VCE software version 5.1.2 (Groeneveld et al., 2008).

### 2.3 Model comparison

To compare the linear models described above, the Akaike information criterion (AIC, Akaike, 1973), the Bayesian information criterion (BIC, Schwarz, 1978) and the log-likelihood (LogL) were calculated for each model. The AIC and BIC were defined as follows:

9AIC=2ln⁡L(i)L(0)-(vi-v0),10BIC=2ln⁡L(i)L(0)-(vi-v0)×ln⁡n-rX,

where $v_{i}$ is the number of parameters in the nested alternative model relative to that in model 1, taken as the null model with $v_{0}$ parameters; n is the number of records; and r(X) is the rank of the fixed-effects incidence matrix.

For log-likelihood ( LogL ), the following formula was used:

11 $$-2\mathrm{LogL}=n\left[\log\left(2\pi \right)+\log\left(\mathrm{SSE}/n\right)+1\right].$$

## 3 Results and discussion

The means and standard deviations of TDMY, age in lambing date, suckling period and litter size are presented in Table 4. The average TDMY was 764.47 mL. The range of variation was wide, ranging from 100 to 3875 mL. The coefficient of variation of TDMY was relatively high at 56 %. As expected, the average TDMY of White Maritza sheep (764.47 mL) was lower than that of the specialized dairy sheep breeds of East Frisian (2.33 kg) (Hamann et al., 2004), Asaf Spanish (1660 mL) (Gutierrez et al., 2007), Lacaune dairy sheep (1820 mL) (Hernandez et al., 2011), Valle del Belice (1167 g) (Riggio et al., 2007), Churra (956 mL) (Othmane et al., 2002), Sfakia dairy ewes (0.86 kg) (Volanis et al., 2002) and the Bulgarian dairy synthetic population (0.896 L) (Krastanov et al., 2018). In terms of litter size at birth, compared to the East Frisian sheep breed (2.09) (Hamann et al., 2004), that of the White Maritza sheep was also lower at 1.39. Earlier studies on the litter size of sheep of the White Maritza sheep breed for the period 1991–1999 revealed a litter size of 1.546 (Dimov, 1999), and for the period 2002–2005, Vuchkov (2009) revealed litter size of 1.36. The prolificacy of the White Maritza sheep breed established in this study (1.39) was similar to that of the competing synthetic dairy sheep population in the Plovdiv region at 1.34 (Dimov and Kuzmanova, 2007) and 1.36 (Zhelyazkova et al., 2014). The average age at lambing was 1382.53 d (3.79 years) with high variation from 362 to 3729 d. The age of the sheep at lambing has a significant effect on test day milk yield with high probability (Zhelyazkova and Dimov, 2023).

**Table 4:** Descriptive statistics for the following traits: test day milk yield (TDMY), age at lambing (AgeLam), suckling period (Suck) and litter size.

Traits	n	$\bar{x}$	SD	CV (%)	Min	Max
TDMY, mL	8768	764.47	431.74	56	100	3875
AgeLam, days	2026	1382.53	632.85	46	362	3729
Suck, days	2026	68.10	19.06	28	30	150
Litter size, n	2026	1.39	0.49	35.25	1	2, >2

SD represents the standard deviation, and CV represents coefficient of variation.

**Table 5:** Raw means and standard deviations of TDMYs and DIMs depending on the sequence of test days for the White Maritza sheep breed and the relative share of the records for the test days.

Sequence of test days	Records	TDMYs (mL)	
	n	$\bar{x}$	± SD
1	2026	1074.34	493.10
2	2026	872.25	407.46
3	2026	682.01	323.21
4	1507	560.04	285.40
5	817	472.65	230.00
6	326	412.62	199.30
7	34	332.54	167.29
8	6	233.33	121.11
Total	8768	764.47	431.74

SD represents the standard deviation.

The database structure shows that, together, the first three test day milk records of ewes represent 69.32 % of the total TDMY records (8768) for the entire database (Table 5). The decrease in the number of records after the third test day for ewes is due to the different durations of the milking periods of different ewes in different flocks. The differences in the lengths of the milking periods in ewes are due to several reasons: long lambing campaigns and the individual abilities of the ewes and the specific situations of each farm in relation to the sale of milk. In accordance with our expectations, the highest milk yield was on the first test day (1074.34 mL). Gradually, milk yield reduced up until to the eighth test day. The milk yield of the ewes was reduced by half on the fifth test day (472.65 mL). The share of test day records received on the eighth test day was non-significant (0.07 %). The relatively high value of the standard deviation in the classes was an indication of a large variation in the test day milk records.

**Table 6:** Additive genetic variance ( $\sigma_{a}^{2}$ ), flock–year–test-day variance ( $\sigma_{\mathrm{ftd}}^{2}$ ), permanent environmental variance ( $\sigma_{\mathrm{pe}}^{2}$ ), residual variance ( $\sigma_{e}^{2}$ ), heritability ( $h^{2}$ ) and repeatability ( $r_{w}$ ) coefficients of test day milk yield using REP models (REP 1, 2 and 3) for the White Maritza sheep breed calculated as a ratio between the general phenotypic variance (taking into account the animal age in terms of lambing day for the White Maritza sheep breed).

REP Models	$\sigma_{a}^{2}$ ± SE	$\sigma_{\mathrm{pe}}^{2}$ ± SE	$\sigma_{\mathrm{fytd}}^{2}$ ± SE	$\sigma_{e}^{2}$ ± SE	$h^{2}$ ± SE	$r_{w}$ ± SE
REP 1	0.355 ± 0.060	0.029 ± 0.048	0.289 ± 0.021	0.327 ± 0.012	0.355 ± 0.060	0.384 ± 0.045
REP 2	0.344 ± 0.047	0.032 ± 0.042	0.278 ± 0.019	0.346 ± 0.012	0.344 ± 0.047	0.376 ± 0.051
REP 3	0.347 ± 0.060	0.031 ± 0.049	0.298 ± 0.020	0.324 ± 0.011	0.347 ± 0.060	0.379 ± 0.065

It is obvious that all three tested REP models estimate variance components without significant differences. The non-significant differences observed in the evaluation of additive variance components highlight some of the scarcely noticeable trends that are likely to be more pronounced with a larger database volume. The significance of the lactation stage expressed by DIM3 or DIM (Zhelyazkova and Dimov, 2023) and its effect on daily milk yield during lactation were necessary to be included either as a fixed effect (REP model 1) or as a linear regression (REP model 2). REP model 1 is a classic variant of a mixed linear model in which the stage of lactation (DIM3) is separate as a fixed effect at 3 d intervals starting from the 30th day of lactation.

It can be seen in Table 6 that REP model 1 with the described random effects explains just over half of the phenotypic diversity, and the heritability has a logical mean value of 0.355 ± 0.060.

Replacing DIM3, a fixed effect, with DIM, described as a linear regression in the parity effect, in REP model 2 shows a barely noticeable trend of a slightly increased evaluation of the residual variance ( $\sigma_{e}^{2}=0.346$ ) and also of a lower assessment of the flock–year–test-day factor ( $\sigma_{a}^{2}=0.278$ ).

The inclusion of the Ali–Schaeffer regression in REP model 3 helps to highlight the influence of the flock–year–test-day effect ( $\sigma_{\mathrm{fytd}}^{2}=0.298$ ) and to decrease the residual variance ( $\sigma_{e}^{2}=0.324$ ), and it provides approximately the same estimation of the additive component ( $\sigma_{a}^{2}=0.347$ ).

There are no significant differences in the values of $h^{2}$ calculated by the three REP models (Table 6). The same applies to the repeatability coefficients, which, for all three REP models, are around 0.38 with slight differences in the third decimal place. The obtained repeatability coefficients of this study for the three tested REP models were lower compared to those for the Valle del Belice sheep breed, in which Cappio-Borlino et al. (1997) calculated a higher value of 0.45. When the effect of lactation stage (DIM) was included as a fixed effect in REP model 1, the heritability estimate had a tendency to be greater ( $h^{2}=0.355\pm 0.060$ ). Heritability and repeatability coefficients for the test day milk yield of White Maritza sheep calculated by the described three REP models were slightly higher than the same genetic parameters in the Chios sheep breed – 0.23 ± 0.015, as calculated by Ligda et al. (2000) – and were significantly higher than the calculated $h^{2}$ of 0.11 for the Bovek, Improved Bovek and Istrian Parmen sheep breeds (Komprej et al., 2009).

Estimating genetic parameters for the test day milk yield of Bovec, Improved Bovec and Istrian Pramenka sheep breeds, Komprej et al. (2011) also calculated low values for $h^{2}$ of 0.10 to 0.23, depending on the month of lactation. Exploring the genetic trends of daily milk production with TDM, Oravcova and Peskovicova (2008) calculated low values of $h^{2}$ in Tsigai, Improved Valachian and Lacaune sheep breeds – 0.19, 0.10 and 0.15, respectively.

The heritability and repeatability coefficients presented in Table 6 are similar to those obtained by Jawasreh and Khasawneh (2007) for the Awassi sheep breed reared in Jordan, with $h^{2}$ from 0.26 to 0.27 and $r_{w}$ from 0.27 to 0.34. Obviously, the potential of REP models to discover and estimate genetic diversity in different sheep breeds is limited to the range of 0.10–0.35.

In addition, the use of repeatability models is associated with the following assumption: during the lactation period, the environmental variance and the genetic correlations between test day milk yields at different stages of lactation are constant values (Ptak and Schaeffer, 1993).

Considering some assumptions and disadvantages of the REP models, we attempted to construct and test three RRMs in the hope of estimating higher values of the heritability of milk yield in the White Maritza sheep breed.

In this regard, based on the REP model 1, three models with random regression RRM1, RRM2 and RRM3 were constructed, associated with the use of first-, second- and third-order polynomials (both for the random effects of the animal and the permanent environment).

**Figure 1:**
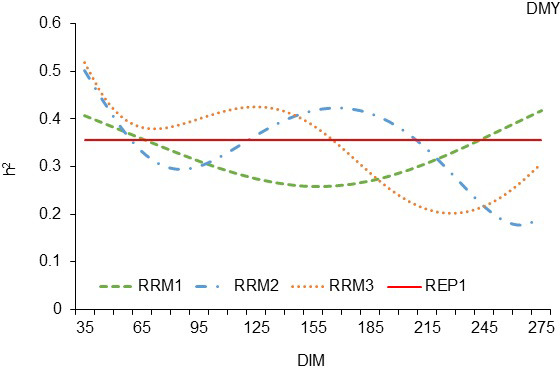
Estimation of heritability trajectories of TDMY depending on the DIM in the test day milk yields of the first, second and third linear polynomials, with fixed linear regressions for the sheep age in terms of the lambing day for White Maritza sheep breed.

The trajectories of $h^{2}$ calculated by the three RRMs were not similar but instead demonstrated some differences, both at the beginning and in the middle of the milking period (Fig. 1).

At the beginning of the milking period (until the 65th day of lactation), all three random regression models showed higher heritability estimates than REP model 1. The RRM3 model led to higher heritability estimates until the 165th day of lactation. After the 165th day of lactation, the trajectory of $h^{2}$ calculated by RRM with the third-degree polynomial (RRM3) remained lower than the straight line of REP model 1 until the end of the lactation period. The trajectories of $h^{2}$ calculated with the other two regression models (RRM1 and RRM2) were lower than the trajectory of model RRM3, which means that regression models with first- and second-degree polynomials were insufficient for calculating higher heritability than REP model 1. In RRM1, most of the trajectory of $h^{2}$ was below the straight line of model REP1, which shows that a random regression model with a polynomial of the first degree was not able to reveal genetic diversity on the basis of milk yield on the test day in the White Maritza sheep breed. The trajectory of $h^{2}$ calculated by RRM2 was too unstable during the milking period, and very little part of it exceeded the straight line of REP model 1.

**Figure 2:**
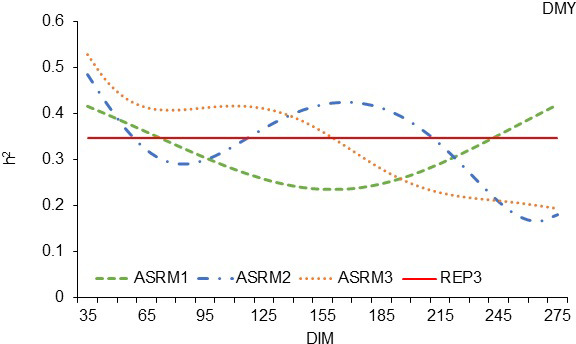
Estimation of heritability trajectories of TDMY depending on the DIM in the test day milk yields, with ASRMs of first-, second- and third-order linear polynomials and with fixed linear regressions for the age in terms of lambing day for the White Maritza sheep breed.

The trends in the trajectories of $h^{2}$ calculated by the three tested ASR models (Fig. 2) were similar to those calculated with random regression models (Fig. 1). Despite the similar trends, a more pronounced superiority of the ASRM3 could be observed, and a higher value of $h^{2}$ could be calculated. In Fig. 2, it can be seen that, again, an ASRM with a polynomial of the third degree calculates a higher value of $h^{2}$ until the 165th day of lactation. The heritability estimates of 0.528 in the beginning of the milking period are gradually reduced to 0.190 by the end of lactation, which is a similar range to that reported by Horstick et al. (2002), but the trajectories of $h^{2}$ are different because, in our cases, heritability estimates decrease to the end of lactation, unlike with the cited authors, where, in their case, the heritability estimates increase to the end of lactation. The trajectories of $h^{2}$ calculated by the three tested ASR models in this analysis were very different in comparison to the trajectory of heritability estimates of the milk yield of Mursiano–Granadina goats, where the trajectory was very flat (Menéndez-Buxadera et al., 2010). Mucha et al. (2014) also reported a different trajectory of heritability estimates in dairy goats because the authors established the highest heritability estimates in the middle of lactation between 200 and 250 d of milking.

The trajectories of $h^{2}$ shown in Figs. 1 and 2 show that random regression models of the first and second order of polynomials cannot reveal more genetic diversity than repeatability model 1. During most of the milking period, the models RRM1 and ASRM1 with first-order polynomials calculated $h^{2}$ values lower than those of REP model 1 and REP model 3, respectively.

The ability of a model to reveal greater genetic diversity depends on the properties of the model. However, the conditions under which the measurements of milk yield were made on the test day have a significant impact. In fact, this is the advantage of the test day models that take into account the environmental effects on the test day. Therefore, the data structure must reflect the environmental conditions as much as possible. This requires the registration of detailed data regarding the conditions under which the milk yield was measured.

The change in the trajectories of $h^{2}$ in the different random regression models in the case of the White Maritza sheep breed can be explained by the fact that the first test day and sometimes the second test day were carried out during the indoor rearing period, and the other test days were carried out when the sheep were grazing. The feeding and rearing conditions during the indoor rearing period for most of the flocks were relatively the same. Grazing sheep are a prerequisite for the estimated phenotypic diversity to be due more to the environment than to genetic causes. In order to differentiate that part of the residual variance that is due to the diet and rearing regime (indoor feeding or grazing), it is necessary to indicate that the record on the test day in terms of the accumulation of test day milk yield data should be noted in addition to the regime of feeding and rearing (indoor feeding or grazing). In our opinion, this could further reduce the residual variance. Calculating a higher value of $h^{2}$ and further higher breeding values of animal candidates for the continuation of the next generation of animals is key to the success of the breeding program for the genetic improvement of milk yield in sheep.

Considering the three RRMs tested in this analysis, which were extended on the basis of REP model 1, it can be noted that the trajectory of $h^{2}$ calculated through the RRM3 model exceeded the trajectories of $h^{2}$ calculated by the other two random regression models, RRM1 and RRM2 (Fig. 1), but RRM3 had higher estimates of LogL, AIC and BIC (Table 7), which means that the three tested random regression models were less well fitted than REP model 1.

**Table 7:** Compare the log-likelihood (LogL), Akaike information criterion (AIC) and Bayesian information criterion (BIC) to select the best model.

Models	LogL	AIC	BIC
REP1	1503.45	1511.45	1518.03
REP2	1852.99	1860.99	1867.57
REP3	1113.54	1121.54	1128.14
ASRM1	2029.32	2045.32	2060.74
ASRM2	2320.39	2348.39	2377.74
ASRM3	3088.99	3132.99	3181.79
RRM1	2419.23	2435.23	2450.64
RRM2	2701.69	2729.69	2759.03
RRM3	3472.75	3516.75	3565.53

REP1, 2 and 3 represent the repeatability models; ASRM1, 2 and 3 represent the Ali and Schaeffer regression models; RRM1, 2 and 3 represent random regression models.

The Akaike information criterion (AIC) is an estimator of prediction error and thereby the relative quality of statistical models for a given set of data (Stoica and Selen, 2004; McElreath, 2016; Taddy, 2016). Ideally, the use of AIC should be concurrent with the use of BIC; there are very subtle theoretical differences between the two criteria, and their only difference in practice is the size of the penalty. Given the selected models, to process the data, AIC evaluates the quality of each model relative to each of the other models; thus, AIC provides a model selection tool. BIC and LogL are also used for a similar purpose.

Table 7 presents the AIC, BIC and LogL of the tested models. Since the comparison of models with the same fixed effects is possible, a comparison of REP1 with RRM1, RRM2 and RRM3 was performed. The comparison indicates that, in the available database used in this study, REP model 1 was a more well-fitted model compared to the three RRMs. In the second type of comparison of REP2 with REP3 and all the ASRMs, REP3 was the best-fitted model because all three criteria had the lowest values and defined REP3 as the best fit model.

## 4 Conclusions

Although the noted advantages of random regression models revealed, to a great extent, the genetic diversity of test day milk yield, at this stage, under the specific environmental conditions and the available database, repeatability models achieved a better balance between complexity and fitness and a smaller prediction error compared to random regression models and Ali–Schaeffer regression models.

It could be concluded that the most suitable models for estimating the heritability of test day milk yield are the reputability model, with fixed effects of days of milking, and the repeatability model, with the Ali–Schaeffer fixed regression through the lambing sequence.

## Data Availability

The data are available upon reasonable request from the corresponding authors.
